# Reframing the link between metabolism and NLRP3 inflammasome: therapeutic opportunities

**DOI:** 10.3389/fimmu.2023.1232629

**Published:** 2023-07-20

**Authors:** Miguel A. Ortega, Diego De Leon-Oliva, Cielo García-Montero, Oscar Fraile-Martinez, Diego Liviu Boaru, Amador Velazquez de Castro, Miguel A. Saez, Laura Lopez-Gonzalez, Julia Bujan, Miguel Angel Alvarez-Mon, Natalio García-Honduvilla, Raul Diaz-Pedrero, Melchor Alvarez-Mon

**Affiliations:** ^1^ Department of Medicine and Medical Specialities, Faculty of Medicine and Health Sciences, University of Alcalá, Alcalá de Henares, Spain; ^2^ Ramón y Cajal Institute of Sanitary Research (IRYCIS), Madrid, Spain; ^3^ Pathological Anatomy Service, Central University Hospital of Defence-University of Alcalá (UAH) Madrid, Alcala de Henares, Spain; ^4^ Department of Surgery, Medical and Social Sciences, Faculty of Medicine and Health Sciences, University of Alcalá, Alcala de Henares, Spain; ^5^ Department of Psychiatry and Mental Health, Hospital Universitario Infanta Leonor, Madrid, Spain; ^6^ Department of General and Digestive Surgery, University Hospital Príncipe de Asturias, Madrid, Spain; ^7^ Immune System Diseases-Rheumatology and Internal Medicine Service, University Hospital Príncipe de Asturias, CIBEREHD, Alcalá de Henares, Spain

**Keywords:** NLRP3 inflammasome, pyroptosis, mitochondrial dysfunction, mtROS, metabolic regulation

## Abstract

Inflammasomes are multiprotein signaling platforms in the cytosol that senses exogenous and endogenous danger signals and respond with the maturation and secretion of IL-1β and IL-18 and pyroptosis to induce inflammation and protect the host. The inflammasome best studied is the Nucleotide-binding oligomerization domain, leucine-rich repeat-containing family pyrin domain containing 3 (NLRP3) inflammasome. It is activated in a two-step process: the priming and the activation, leading to sensor NLRP3 oligomerization and recruitment of both adaptor ASC and executioner pro-caspase 1, which is activated by cleavage. Moreover, NLRP3 inflammasome activation is regulated by posttranslational modifications, including ubiquitination/deubiquitination, phosphorylation/dephosphorylation, acetylation/deacetylation, SUMOylation and nitrosylation, and interaction with NLPR3 protein binding partners. Moreover, the connection between it and metabolism is receiving increasing attention in this field. In this review, we present the structure, functions, activation, and regulation of NLRP3, with special emphasis on regulation by mitochondrial dysfunction-mtROS production and metabolic signals, i.e., metabolites as well as enzymes. By understanding the regulation of NLRP3 inflammasome activation, specific inhibitors can be rationally designed for the treatment and prevention of various immune- or metabolic-based diseases. Lastly, we review current NLRP3 inflammasome inhibitors and their mechanism of action.

## 1 Introduction

The innate immune system plays a crucial role in protecting the body against various pathogens and maintaining tissue homeostasis. In recent years, the relationship between the innate immune system, inflammation, metabolism, and metabolic diseases has gained significant attention (1–4). It is now recognized that dysregulation of metabolic processes can profoundly impact immune responses and contribute to the development of metabolic diseases, such as obesity, type 2 diabetes, and cardiovascular disorders (5–7).

In this context, inflammasomes, multiprotein signaling platforms, have emerged as critical players linking metabolism and inflammation (8, 9). Inflammasomes are multiprotein signaling platforms that detect exogenous and endogenous danger signals in the cytosol and respond activating caspase-1 which leads to production and release of proinflammatory cytokines IL-1β and IL-18 (10). Likewise, inflammasome is capable of inducing pyroptosis (also known as inflammatory cell necrosis), a specialized form of programmed cell death that is triggered by inflammatory signaling pathways characterized by the rapid and lytic cell death accompanied by the release of pro-inflammatory cytokines and cellular contents (11). Therefore, inflammasomes are an important component of innate immunity that ensure protective responses against pathogens and tissue homeostasis (12–14). In addition to their role in detecting danger signals, inflammasomes have been implicated in metabolic dysfunction, insulin resistance, and the development of metabolic diseases. The activation of inflammasomes can disrupt insulin signaling, promote chronic low-grade inflammation, and contribute to metabolic imbalances (15–18). Moreover, inflammasomes have recently been linked to others programmed cell deaths (PCD), besides pyroptosis, including apoptosis, necroptosis, PANoptosis and ferroptosis (19–23). PCD is an essential process that preserve tissue homeostasis, eliminate damaged cells and regulate immunological responses (24, 25). Apoptosis, necroptosis, PANoptosis and ferroptosis have drawn the most interest because of their consequences in both physiological and medical conditions. Recent studies have shown the complex interaction between the inflammasome and these programmed cellular deaths, highlighting how this interaction affects inflammatory reactions and disease progression (19).

There exist different types of inflammasomes. Nucleotide-binding oligomerization domain (NOD), leucine-rich repeat-containing (LRR) (NLR) family pyrin domain containing 3 (NLRP3) inflammasome is the most studied. NLRP3 mutation have been associated with cryopyrin-associated periodic syndrome (CAPS), a group of autoinflammatory disorders characterized by recurrent fevers and systemic inflammation (26).

This review aims to provide a comprehensive overview of the current understanding of the interplay between metabolism and NLRP3 inflammasome activation. We will delve into the molecular mechanisms through which metabolic factors influence NLRP3 inflammasome signaling, emphasizing the role of mitochondrial dysfunction-mitochondrial reactive oxygen species (mtROS) production, metabolites and metabolic pathways in this process. Furthermore, we will explore the therapeutic opportunities that arise from targeting metabolic pathways to modulate NLRP3 inflammasome activation, offering potential strategies for the development of novel anti-inflammatory therapies.

## 2 Structure and assembly of the NLRP3 inflammasomes

**Figure 1:**
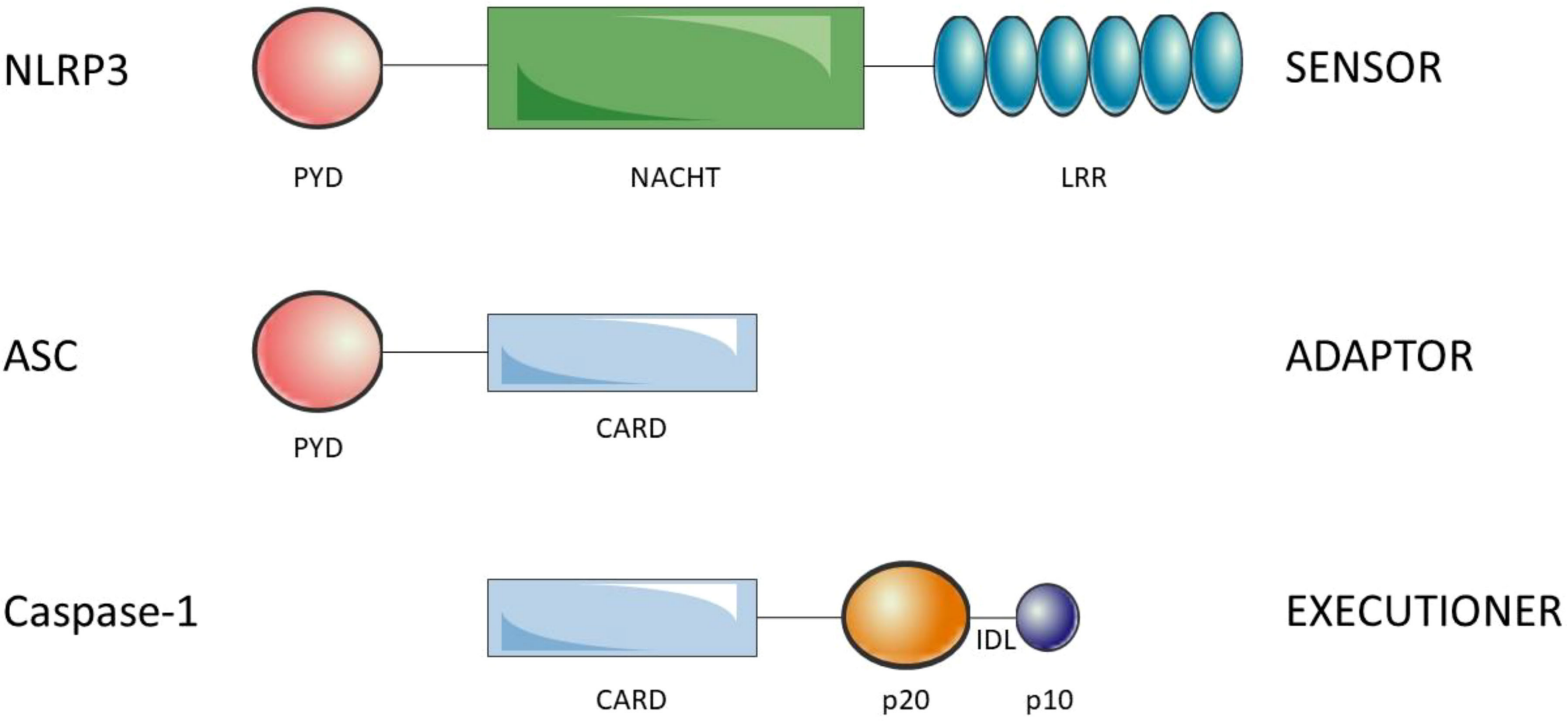
Structure of the components of the NLRP3 inflammasome. NLRP3 is composed of the N-ter PYD, NACHT and C-ter LRR. ASC is composed of theN-ter PYD and the C-ter CARD. Finally, caspase-1 is composed of the N-ter CARD, p20 (large catalytic subunit) and C-ter p10 (small catalytic subnit). The interdomain linker (IDL) binds p20 and p10 and is cleaved to process inactive zymogen pro-caspase-1 to active caspase-1. Oligomerization of NLRP3 and homotypic interactions PYD-PYD and CARD-CARD lead to the NLRP3 inflammasome assembly.

Inflammasomes are composed of the sensors/receptors of the danger signals, the adaptor and the effector. There are a variety of sensors/receptors that classify the distinct inflammasomes which are activated by different stimuli: NLRP1, NLRP3, NLRC4, AIM2 or pyrin (27). The adaptor protein is the apoptosis-associated speck-like protein containing a CARD (ASC) that recruits and activates pro-caspase-1 (28). Finally, the effector is the pro-caspase-1 which is activated by cleavage into caspase-1 (29). NLRP3 is a 1036 amino acid protein that contains three domains, an amino (N)-terminal pyrin domain (PYD), a central NACHT domain and a carboxy (C)-terminal LRR. PYD (3–91) consists of six antiparallel α-helix, is a member of the death domain-fold superfamily and mediates the supramolecular complex formation, probably due to its ability of dimerization (30). The NACHT (NAIP, CIITA, HET-E, TP1) domain (131–649) is responsible for the oligomerization and presents ATPase activity. Is subdivided into FISNA (131–218), NBD (219–372), HD1 (373–434), WHD (435–541) and HD2 (542–649) domains (31). The LRR domain (650-1036) at C-terminal is involved in the activation and ligand sensing of the NLRP3 inflammasome (32, 33) and binds a NIMA-related kinase 7 (NEK7), an essential mediator of NLRP3 activation (34). ASC presents an N-terminal PYD and a C-terminal CARD which allow the interaction with NLRP3 and pro-caspase-1, respectively (35) (see **Figure 1**). Indeed, ASC oligomerization, by assembling into filaments, leads to a supramolecular complex formation, ASC speck, that generates numerous potential sites for caspase-1 activation and serves as an amplification signal (36–38). Caspase-1 is synthesized as an inactive zymogen called pro-caspase-1, which presents an N-terminal CARD, a central p20 catalytic subunit and a C-terminal p10 subunit (39). Pro-caspase-1 is recruited through homotypic interactions of their CARDs with CARDs of ASC. The clustering of individual pro-caspase-1 monomers within the inflammasome triggers proximity-induced dimerization, which leads to the formation of protease active sites, with two active sites per dimer. Then, pro-caspases-1 are autoprocessed by cleavage of interdomain linker, between p20 and p10, to become fully active (40).

## 3 Functions of NLRP3 inflammasome

NLRP3 canonical function consists in the sensing of danger signals, pathogen-associated molecular patterns (PAMPs) and damage-associated molecular patterns (DAMPs), in the cytoplasm of cells as a pattern recognition receptor (PRR) of the innate immune system and respond with the activation and assembly of NLRP3 inflammasome (41). NLRP3 inflammasome activates caspase-1 which drives the cell death by pyroptosis and maturation and release of IL-1β and IL-18 (42). Non canonical functions of NLRP3 are independent of NLRP3 inflammasome and will not be discussed in this review, including TGF-β signaling and R-Smad activation in epithelial cells (43), transcriptional regulator of driving T helper type 2 (TH2) polarization in CD4+ T cells (44) and regulation of apoptosis in epithelial cells trough a conserved non-canonical platform for caspase-8 activation (45, 46).

### 3.1 Pyroptosis

Pyroptosis is a form of programmed cell death that is characterized by a highly inflammatory response. It is mediated by inflammatory caspases, which are caspase-1 in human and mice, caspases-4 and -5 in human and the ortholog caspase-11 in mice (47). NLRP3 inflammasome participates in the canonical and noncanonical inflammasome pyroptotic pathways. The former is driven by the recognition of dangerous signals by the NLRP3 inflammasome and the subsequent activation of caspase-1, which cleaves gasdermin D (GSDMD) at D275 and pro-IL-1β and pro-IL-18 (48, 49). The N-terminal fragment of GSDMD forms a transmembrane pore that disrupts ion and water equilibrium and secretes IL-1β and IL-18, resulting in cell death and inflammatory response (50). The latter is initiated by the recognition and binding of caspases 4/5/11 with lipopolysaccharide (LPS) of Gram-negative bacteria, and their oligomerization and activation (51). Next, GSDMD is cleaved by caspases 4/5/11 and the impairment in ion equilibrium, probably potassium efflux, activates the canonical NLRP3 inflammasome (52). GSDMD transmembrane pores are the mediators of pyroptosis and result in the release of cytosolic content, including PAMPS, DAMPS, IL-1β, IL-18, alarmins, to the extracellular space, alteration of water and ion equilibrium, cell swelling and in some cases the lysis of the cell (50). The major established physiological functions of pyroptosis cell death are host defense against microbial infections (53) and regulation of inflammation (54), and exhibits close associations with a range of conditions, including diseases of the nervous system, infectious diseases, autoimmune disorders, cardiovascular conditions, and tumorigenesis (55).

### 3.2 Maturation and secretion of IL-1β and IL-18

The activated form of caspase-1 cleaves pro-IL-1β and pro-IL-18 at D116 and D36, respectively (56). IL-1β and IL-18 are two members of the IL-1 family. They act through binding with their respective receptors. The secretory mechanism described are through GSDMD transmembrane pores and through membrane rupture and lysis (57). IL-1β and IL-18 play important roles in inflammation (58–60), innate and adaptive immunity (61), bone metabolism (62), central nervous system (CNS) function (63, 64) and metabolism (65–67). Due to their pleiotropic effects, both ILs are involved in several diseases (68, 69).

Despite the mechanism is less well understood, NLRP3 inflammasome is also involved in the secretion of IL-1α (70, 71), high mobility group box 1 (HMGB1) (72, 73), M1 macrophage polarization (74) and modulation of glycolysis (75).

## 4 Activation and regulation of inflammasome

**Figure 2:**
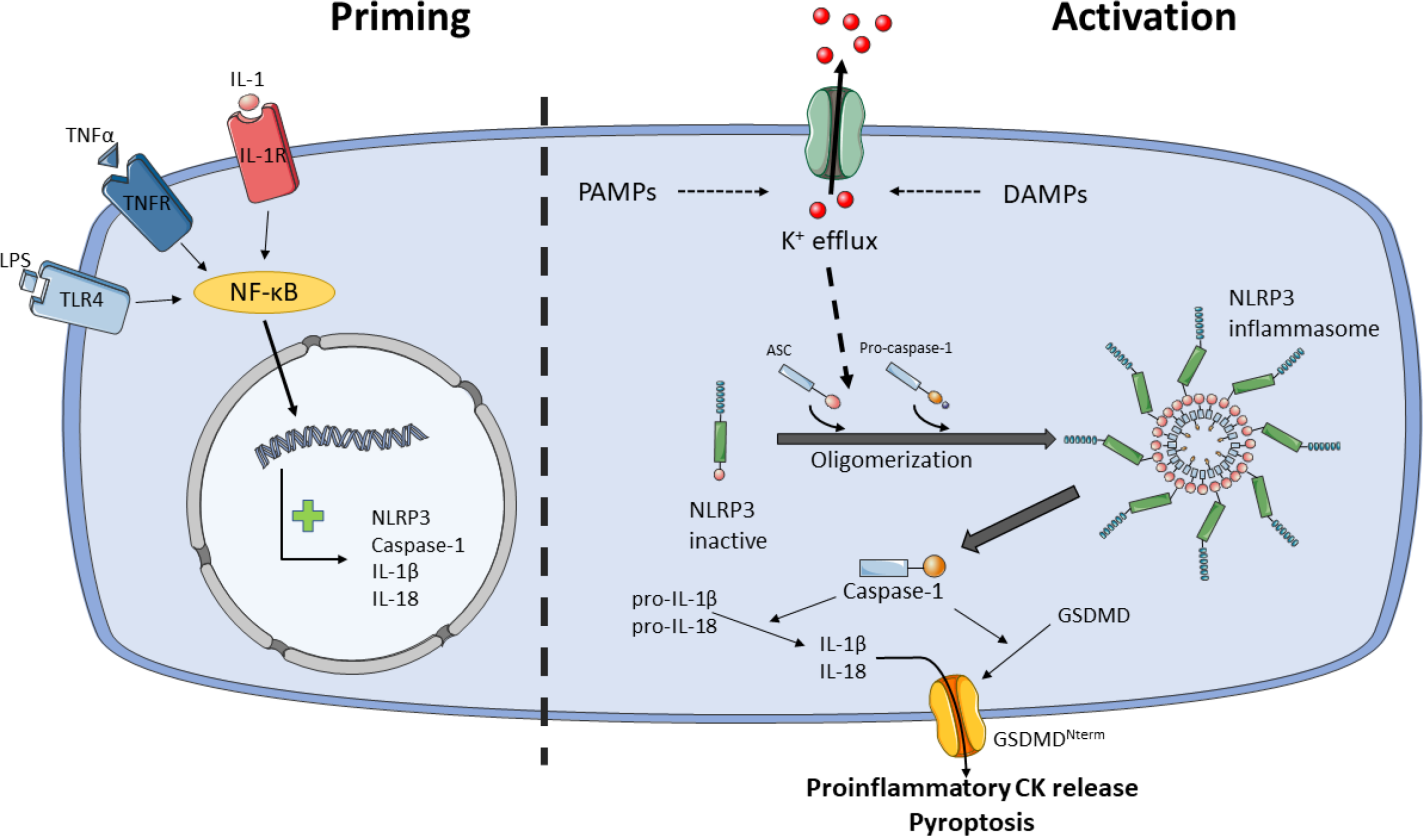
Model for NLRP3 activation by two-step process. Priming (step 1) licenses the cell for NLRP3 inflammasome activation. Certain PAMPs and cytokines (CK) bind to their receptors, e.g.: LPS/TLR4, TNF-α/TNFR or IL-1/IL-1R, leading to activation of NF-κB, which translocate into the nucleus and activates transcription of NLRP3, caspase-1, IL-1β and IL-18. Activation (step 2) starts with the sensing of danger signals, PAMPs and DAMPs, by NLRP3. These stimuli trigger the activation of NLRP3 inflammasome through K^+^ or Cl^-^ efflux, Ca^2+^ flux, lysosomal disruption, mitochondrial dysfunction and reactive oxygen species (ROS) generation, metabolic signals and *trans*-Golgi disassembly, and it seems that K^+^ efflux is the convergence point for the activation of NLRP3 inflammasome. NLRP3 inflammasome activation occurs through NLRP3 and ASC oligomerization and assembly. NLRP3 inflammasome recruits and activates pro-caspase-1. Caspase-1 cleaves and activates pro-IL-1β and pro-IL-18 and GSDMD. Proinflammatory CKs are released through GSDMD pore and pyroptosis. Ultimately, NLRP3 inflammasome signals for inflammation and cell death by pyroptosis.

The activation of NLRP3 inflammasome is well regulated because it is activated in a two-step process, the priming and the activation steps (76) (see **Figure 2**). Likewise, post-translational modifications (PTMs) and multiple NLRP3-interacting protein partners also modulate NLRP3 inflammasome activity at both phases (77). The priming step leads to the transcription of NLRP3, caspase-1, IL-1β and IL-18, despite IL-18 is constitutively expressed, and it is believed that licenses the cell to rapidly responds to activity stimuli. The priming signals are PAMPs/DAMPs detected by PRRs, such as LPS/TLR4 or muramyl dipeptide (MDP)/NOD2, and cytokines that bind to their receptors, such as tumor necrosis factor-α (TNF-α)/TNFR or IL-1β/IL-1R. These signals activate nuclear factor-κB (NF-κB) transcription, which upregulates NLRP3, pro-IL-1β and pro-caspase-1 (41, 78).

Following priming step, recognition of NLRP3 activators leads to oligomerization, assembly and activation of NLRP3 inflammasome. While most PRRs detect one or two PAMPs or DAMPs, the NLRP3 inflammasome stands out for the wide variety of stimuli of different nature it recognizes, including PAMPs such as pore-forming toxins, LPS, viral RNA, fungal β-glucans or DAMPs such as ATP, cholesterol/monosodium urate crystals, alum or silica (79). Due to the diverse nature of these stimuli is unlikely that NLRP3 interacts with all of them. It is currently accepted that cellular stress induced by these stimuli trigger the activation of NLRP3 inflammasome through K^+^ or Cl^-^ efflux, Ca^2+^ flux, lysosomal disruption, mitochondrial dysfunction and reactive oxygen species (ROS) generation, metabolic signals and *trans*-Golgi disassembly (79–81), and it seems that K^+^ efflux is the convergence point for the activation of NLRP3 inflammasome (82–84). However, other levels of regulation have been described such as epigenetic regulation by microRNAs (85, 86), subcellular localization (87), crosstalk between the gut microbiota and NLRP3 is involved in signaling in the gut-brain axis (88), exosomes (89), acute-phase proteins (90) or neuropeptide Calcitonin Gene-Related Peptide (CGRP) (91).

PTMs of NLRP3 inflammasome affect mainly the NLRP3, but also ASC and caspase-1 and promotes or inhibits NLRP3 inflammasome activity depending on the specific modified amino acid residue (77). PTMs include ubiquitination/deubiquitination, phosphorylation/dephosphorylation, acetylation/deacetylation, SUMOylation and nitrosylation (92, 93). The research of these PTMs and the signaling pathways are of great interest in the search for drugs that modulate NLRP3 inflammasome activity in different diseases in which it is deregulated.

Several NLPR3 protein binding partners modulate both priming and activation via domain-domain interactions with NLRP3, ASC or caspase-1. Positive modulators are GBP5, MARK4, HSP90, TXNIP, NEK7, DDX3X, MAVS, MFN2, PKR and MIF, while negative modulators include COPs, POPs, GNB1, SHP, PPARγ, HSP70 and RACK1 (81, 94–96).

It is worth to mention a less-understood alternative NLRP3 activation pathway in human and porcine monocytes and murine dendritic cells (DCs) that only requires signal 1 to activate NLRP3 inflammasome via TLR4-TRIF-RIPK1-FADD-CASP8 signaling upstream of NLRP3, leading to IL-1β secretion but no to pyroptosis (97, 98).

Lastly, pathogenic pollutants and cigarette smoke activate NLRP3 inflammasome via ROS and mtROS generation, contributing to chronic inflammation and fibrosis in the respiratory system, which plays a crucial role in the pathogenesis of respiratory diseases such as asthma, chronic obstructive pulmonary disease (COPD) and lung fibrosis (99). Fine particulate matter (PM2.5) is a major component of air pollution. Exposure to PM2.5 resulted in lung injury in mice, characterized by the infiltration of inflammatory cells and structural abnormalities in the alveoli and upregulation of NRP3 inflammasome (100). ROS production induced by PM2.5 is linked to ATP alteration (101). NLRP3 inflammasome is activated by PM2.5 trough lysosomal damage and cathepsin B release and induce fibrosis in mouse lung (102). Asbestos and crystalline silica also induce NLRP3 inflammasome activation through ROS production (99). BV-2 and HMC-3 microglial cell lines subjected to oxygen–glucose deprivation and reoxygenation with PM2.5 exposure show decreased cell viability, increased NLRP3 inflammasome activation and ROS production (103). Similarly, ozone-induced lung inflammation in murine model activates NLRP3 trough mtROS production (104, 105).

The role of cigarette smoke (CS) in the activation of NLRP3 inflammasome remains controversial. On the one hand, constituents of CS are involved in the activation of NLRP3 through oxidative stress, mitochondrial dysfunction MyD88/NF-κB, HMGB1, endoplasmic reticulum stress, lysosomal destabilization and TLR/NF-κB signaling pathway (106–108). On the other hand, Buscetta et al. found that CS inhibited the expression of NLRP3 at the transcriptional level, but activated caspase-1 in an NLRP3-independent manner (109). Moreover, CS increases NLRP3 ubiquitin-mediated proteasomal processing (110).

Because the aim of this review is to connect the inflammasome activity and metabolism, we will take a closer look at the regulation by mitochondrial dysfunction-ROS generation and metabolic signaling.

### 4.1 NLRP3 inflammasome in mitochondrial dysfunction-mtROS production

The mitochondria are the organelles in charge of adenosine triphosphate (ATP) generation. However, it also regulates cellular processes, including the cell death by apoptosis, through the intrinsic pathway (111). It is considered the connection between the innate immunity and metabolism (112). Moreover, mitochondrion is an important mediator of NLRP3 inflammasome activation as shown in the multiple regulatory mechanisms where it is involved and inhibition of electron transport chain prevents NLRP3 inflammasome activation (113). The best-recognized mechanisms are the increased generation of mitochondrial ROS (mtROS) during cellular stress (114), the release of oxidized mitochondrial DNA (mtDNA) into the cytosol (115) and the recruitment of NLRP3 through cardiolipin (116). Furthermore, multiple NLRP3 signaling activators lead to mitochondrial dysfunction, which is associated with the pathogenesis of metabolic diseases (99, 117, 118).

mtROS are increased during cellular stress and mediated through voltage dependent anion-selective channel (VDAC) (100). Inhibition of mitochondrial Complexes I and III induce mtROS and NLRP3 activation, independent of K^+^ efflux (101, 102, 114). However, Muñoz-Planillo et al. showed that ROS production is dispensable for NLRP3 activation (82) and the nuclear factor erythroid 2-related factor 2 (NRF2), a regulator of redox homeostasis, has been described as both activator (103) and inhibitor (119) of NLRP3 inflammasome.

Likewise, mtDNA binds to and activates NLRP3 inflammasome (115, 120). According to Nakahira et al, mtDNA is released into the cytoplasm through mitochondrial permeability transition (MPT) pores in a mtROS-dependent manner (101). Moreover, TLR2, TLR3 or TLR4 agonists increase mtDNA synthesis through upregulation of IRF1-CMPK2, allowing activation of the NLRP3 inflammasome (121). In line with the above mentioned, mitophagy reduces mtROS and mtDNA and thus, activation of NLRP3 inflammasome, and inhibitors of mitophagy achieve the opposite effect (114).

Lastly, when mitochondrial cardiolipin (mtCL), a unique phospholipid of the inner membrane (IMM), translocate to the outer membrane (OMM) binds the LRR domain of NLRP3 (122). Recently discovered, mtCL recruits IL-1α through a similar CL binding domain present in LC3b, a CL binding protein that triggers mitophagy (123). Both lead to NLRP3 inflammasome activation. The mitochondrial antiviral signalling protein (MAVS), in complex with mitofusin 2, recruits NLRP3 to the mitochondria during RNA viral infections. Subramanian et al. identify an N-terminus sequence in NLRP3 that associates with MAVS. Park et al. suggest that putting close NLRP3 to mtROS may promote its oligomerization and activation. However, MAVS does not seem essential for NLRP3 inflammasome activation (124, 125). Lastly, to demonstrate the importance of subcellular localization, NLRP3 associates to the endoplasmic reticulum (ER) in inactivated cells. Then, activators of NLRP3 inhibit Sirtuin2, and through acetylated α-tubulin via dynein-dependent mitochondrial transport, ASC on mitochondria is apposed to NLRP3 (126). Its assembly takes place in mitochondria-associated endoplasmic reticulum membranes (MAMs) in the perinuclear space (87, 126).

### 4.2 NLRP3 in metabolic regulation: metabolites and metabolic pathways

Metabolic reprogramming refers to the alterations in cellular metabolic pathways that occur in immune cells, enabling them to adapt their energy production and biosynthetic processes to support immune responses effectively (127). Indeed, Traba et al. demonstrated that nutrient levels in human subjects modulate NLRP3 inflammasome activation (128). For instance, activated macrophages undergo a metabolic switch to aerobic glycolysis (129, 130). Not only glucose metabolism, but also lipid and purine metabolism and the tricarboxylic acid (TCA) cycle can regulate the activation/inhibition of the NLRP3 inflammasome. **Table 1** presents a detailed view of the principal mechanisms of activation of NLRP3 inflammasome through metabolites and metabolic pathways.

**Table 1:** Summary of the metabolic signals regulating the NLRP3 inflammasome activation.

Metabolic pathway	Metabolite/enzyme	Cell type	Activation/inhibition of NLRP3 inflammasome	Mechanism of action	References
**Glucose metabolism**	Glucose	HUVEC	Activation	HG upregulate ELF3-SET8-MARK4 pathway	(131)
		TEC	Activation	HG upregulate CD36, which inhibits FAO and increase mtROS	(132)
		Macrophages	Inhibition	Under hypoxia, glycolysis is induced and ATP maintains closed the K_ATP_ channels	(133)
	Pyruvate	Macrophages	Inhibition	Ethyl pyruvate inhibits NLRP3 inflammasome activation by preservation of mitochondrial integrity	(134)
		Microglial cells	Inhibition	Ethyl pyruvate suppresses activation of the NLRP3 inflammasome through the regulation of miR-223 and the NF-κB/HMGB1 axis	(135)
	HK1	Macrophages	Activation	mTORC1-HK1-dependent glycolysis	(136)
	HK	Macrophages	Activation	NAG inhibits HK and the complex dissociates from OMM and activates NLRP3 inflammasome	(137)
	G6PD	THP-1	Activation	G6PD-kd decreases NOX-dependent ROS production and inhibits NLRP3 inflammasome	(138)
	GAPDH and α-enolase	Macrophages	Inhibition	Inhibition of GAPDH and α-enolase, resulting in perturbed metabolite production and flux, activates NLRP3 inflammasome	(139)
	Aldolase A	Macrophages	Activation	Aldolase A monitors glycolytic flux and maintains NLRP3 inflammasome activation via AMPK activation	(140)
	PKM2	Macrophages	Activation	PKM2-mediated glycolysis modulates lactate-mediated EIF2AK2 phosphorylation	(141)
	LDH	Macrophages	Activation	Inhibition of LDH reduces response of NLRP3 inflammasome to NLRP3 agonists	(142)
	Amylin	Macrophages	Activation	Phagocytosis of amylin results in ROS generation and MyD88 activation	(143, 144)
**TCA cycle**	Succinate	Synovium	Activation	Succinate accumulation activates NLRP3 inflammasome in a manner HIF-1α dependent	(145, 146)
	Itaconate	Macrophages	Inhibition	Itaconate inhibited NLRP3 and NEK7 interaction	(147)
**Lipid metabolism**	Palmitate and ceramide	Macrophages	Activation	Accumulation of lipids leads to activation of NLRP3 inflammasome via AMPK-autophagy-mtROS signaling pathway	(148, 149)
	BHB	Macrophages	Inhibition	Prevents K^+^ efflux	(150)
	Butyrate and propionate		Inhibition	Inhibit NLRP3 priming and activation by palmitate	(151)
	Cholesterol cristals	Macrophages	Activation	Induce lysosomal destabilization and cathepsin B leakage	(152)
	NOX4	Macrophages	Activation	Upregulates CPT1A enhancing FAO	(153)
	SAA	Macrophages	Activation	Activates NLRP3 inflammasome via P2X7 receptor, cathepsin B-sensitive pathway and ROS production	(154, 155)
**Purine metabolism**	UA		Activation	Via mtROS production	(156)
	ATP	Immune cells	Activation	Activates P2X7 receptor leading to K^+^ efflux	(157)

HUVEC, human umbilical vein endothelial cell; HG, high glucose; ELF3, E74 like ETS transcription factor 3; MARK4, microtubule affinity-regulating kinase 4; TEC, renal tubular epithelial cells; FAO, fatty acid oxidation; mtROS, mitochondrial reactive oxygen species; K_ATP_ channel, ATP-sensitive K+ channels; HK1/2, hexokinase 1/2; mTORC1, mammalian target of rapamycin complex 1; NAG, N-acetylglucosamine; OMM, outer mitochondrial membrane G6PD, glucose-6-phosphate dehydrogenase; G6PD-kd, G6PD knockdown NOX, NADPH oxidase; GAPDH, glyceraldehyde-3-phosphate dehydrogenase; PKM2, pyruvate kinase M2; EIF2AK2, eukaryotic translation initiation factor 2 alpha kinase 2; LDH, lactate dehydrogenase; MyD88, Myeloid differentiation primary response 88; HIF-1α, hypoxia inducible factor-1α; NEK7, NIMA related kinase 7; AMPK, AMP-activated protein kinase; CPT1A, carnitine palmitoyltransferase 1A; BHB, β-hydroxybutyrate; FASN, fatty acid synthase; UCP2, uncoupling protein-2; MSU, monosodium urate; SAA, serum amyloid.

#### 4.2.1 Glucose metabolism and NLRP3 inflammasome activation

First, glucose has been shown to regulate the NLRP3 inflammasome. High glucose (HG) in endothelial cells increases expression of E74 like ETS transcription factor 3 (ELF3) and decreases SET8, a methyltransferase, leading to up-regulation of microtubule affinity regulatory kinase 4 (MARK4) (131). MARK4 activates the NLRP3 inflammasome through a microtubule-dependent mechanism (158). In addition, human renal proximal tubular cells under HG conditions increase CD36 levels, leading to a metabolic shift from fatty acid oxidation to glycolysis, which promotes mtROS production and NLRP3 inflammasome activation (132). However, Watanabe et al. proposed a model of macrophages subjected to hypoxia, in which, under HG conditions, hypoxia induces glycolysis, resulting in intracellular ATP generation and subsequent closure of KATP channels, with the NLRP3 inflammasome remaining inactive (133). On the other hand, hypoxia and glucose deprivation cause a decrease in ATP levels, the opening of KATP and, therefore, the activation of the NLRP3 inflammasome. In this model, mtROS played no significant role in NLRP3 inflammasome activation. Thus, it appears that the NLRP3 inflammasome can recognize the energetic state of the cell and respond.

Pyruvate is a key intermediary metabolite, the end product of glycolysis that can be transformed into acetyl-CoA, lactate or alanine. In experiments, ethyl pyruvate prevents NLRP3 inflammasome activation while preserving mitochondrial integrity. Pyruvate inhibits ATP- and nigericin-induced accumulation of electron-dense mitochondria, maintains mitochondrial membrane potential, and prevents ATP- or nigericin-triggered release of mtDNA into the cytoplasm (134). In microglial cells, ethyl pyruvate demonstrates strong efficacy in suppressing NLRP3 inflammasome activation via regulation of miR-223 and the NF-κB/HMGB1 axis (135).

Hexokinase 1 (HK1) catalyzes glucose entry into glycolysis in the cytosol. In macrophages, NLRP3 inflammasome activation is regulated by mTORC1-induced up-regulation of HK1-dependent glycolysis (136). Furthermore, Wolf et al. propose that hexokinase is an innate immune receptor that when inhibited by N-acetylglucosamine dissociates from the outer mitochondrial membrane and activates the NLRP3 inflammasome (137).

Glucose-6-phosphate dehydrogenase (G6PD) catalyzes the entry of glucose into the pentose phosphate pathway. One of its main functions is to control the redox state of the cell (159). Yen et al. found that in G6PD-deficient individuals, increased susceptibility to pathogens is attributed in part to altered NOX/p38 MAPK/AP-1 redox signaling, leading to negative effects on both inflammasome activation and bactericidal response (138). Inhibition of the glycolytic enzymes GAPDH and α-enolase by the small molecule GB111-NH2, which disrupts glycolytic flux, leads to NLRP3 inflammasome activation (139). Finally, the catalytic enzymes aldolase A, pyruvate kinase M2 (PKM2), lactate dehydrogenase (LDH), and the peptide hormone amylin (secreted by pancreatic β-cells) participate in the activation of the NLRP3 inflammasome in macrophages (140–144).

#### 4.2.2 The tricarboxylic acid cycle and NLRP3 inflammasome activation

Regarding TCA, there are two intermediates that lead to opposite effects in terms of NLRP3 inflammasome activation. On the one hand, hypoxic induction of TGF-β1 in the synovium of rheumatoid arthritis (RA) rats resulted in elevated succinate accumulation. This was caused by inhibition of succinate dehydrogenase (SDH) activation and led to NLRP3 inflammasome activation, which was dependent on HIF-1α induction. Moreover, citrate-derived itaconate has gained attention as an anti-inflammatory modulator in macrophages (160). Hooftman et al. found that itaconate modifies a specific cysteine (C548) in NLRP3 and inhibits its activation by interfering with the binding partner of NEK7 (147). Taken together, dysregulations in the TCA cycle, the central metabolic pathway for carbohydrates, lipids, and proteins, are potential activators of the NLRP3 inflammasome.

#### 4.2.3 Lipid metabolism and NLRP3 inflammasome activation

Lipid metabolism plays a crucial role in a variety of cellular processes, such as energy production, membrane synthesis and signaling pathways. New research has revealed a significant connection between lipid metabolism and NLRP3 inflammasome activation/inhibition. First, the accumulation of fatty acids palmitate and ceramide activate the NLRP3 inflammasome through the AMPK-autophagy-mtROS signaling pathway (148, 149). In contrast, the ketone metabolite β-hydroxybutyrate (BHB) serves as an alternative source of ATP, favoring mammalian survival during periods of energy deficit. BHB blocks the NLRP3 inflammasome by inhibiting K+ efflux (150). In addition, a recent study indicates that butyrate and propionate have inhibitory effects on the priming and activation of the NLRP3 inflammasome by palmitate (151). This inhibition leads to a significant reduction in pro-IL-1β levels, whereas the effect on inflammasome activation itself is relatively modest.

Cholesterol crystals are a common feature of atherosclerotic plaques (161). Cholesterol crystals are phagocytosed by macrophages and stimulate NLRP3 inflammasome activity, probably through lysosomal destabilization (152). Regarding fatty acid oxidation (FAO), deficiency of NADPH oxidase 4 (NOX4) leads to lower expression of carnitine palmitoyltransferase 1A (CPT1A), a key mitochondrial fatty acid transporter. The result is lower FAO and reduced activation of the NLRP3 inflammasome (153). Finally, serum amyloid A (SAA) is an acute-phase protein that increases in serum during inflammation and has a pathogenic role in amyloid A amyloidosis. SAA induces activation of the NLRP3 inflammasome in macrophages through the P2X7 receptor, dependent on cathepsin B activity, but not through lysosomal destabilization, and by generating ROS (154, 155).

## 5 Therapeutic approaches

The recognition of the pivotal role of inflammasomes in the development and progression of metabolic diseases opens up exciting possibilities for therapeutic interventions (9, 162, 163). Targeting inflammasome pathways and modulating their activity holds promise for mitigating inflammation, improving metabolic function, and potentially treating metabolic diseases.

Developing small-molecule inhibitors that specifically target NLRP3 inflammasome components, such as NLRP3 or caspase-1, represents a promising avenue (41). Inhibitors block the activation of the inflammasome complex, thereby reducing the release of proinflammatory cytokines and alleviating inflammation associated with metabolic diseases (164–166). It is important to note that many therapeutic approaches targeting inflammasomes are still in the preclinical or early clinical stages of development. Further research is needed to elucidate the safety, efficacy, and long-term effects of these interventions in human populations. Additionally, considering the complex network of inflammasome-related pathways, combination therapies targeting multiple components may hold even greater potential for effectively managing inflammation and metabolic dysfunction. In the search for potential inhibitors, a broad range of targets can be tested due to the complex signaling cascade that governs NLRP3 inflammasome activation and its functions. For example, TLR4, TNFR and NF-κB signaling of the priming step, activators such as K^+^/Cl^-^ efflux, P2X7 and mtROS, effectors such as GSDMD, interaction binding partners such as NEK7 and, obviously, the NLRP3 inflammasome components NLRP3 receptor, ASC and caspase-1. Currently, the ATPase activity of the NACHT domain in NLRP3 is the target of the majority of NLRP3 inflammasome inhibitors under clinical and preclinical research (see **Table 2**). Despite the existence of drugs targeting IL-1, i.e., canakinumab and anakinra, there is a need in the search of specific inhibitors that target specifically members of the NLRP3 inflammasome. These may intervene at an upstream point in the inflammatory cascade, potentially providing more comprehensive control over the inflammatory response. Moreover, directly inhibiting IL-1β signaling may affect the immune system more broadly and may have side effects. A more focused strategy, however, may be to specifically block the NLRP3 inflammasome, which lowers the chance of systemic immunosuppression while still producing the desired anti-inflammatory benefits.

**Table 2:** Potential inhibitors of NLRP3 inflammasome and their targets.

Target	Drug	Structure	Mechanism of action	Possible diseases	Study Phase (NCT)	Reference
NLRP3	Oridonin	Ent-kaurane diterpenoid	Blocking the interaction between NLPR3 and NEK7 by covalently attaching to Cys273 of NACHT	T2D, GA, and peritonitis	Phase 4 (NCT05130892)	(167)
	Tranilast	Tryptophan metabolite analog	Binds to NACHT inhibiting oligomerization and ASC binding	T2D, GA, and CAPS	Phase 4 (NCT05130892)	(168)
	CY-09	C172 analog	Interacts with Walker A motif to inhibit ATPase activity	CAPS and T2D	Preclinical	(169)
	MNS	Inhibitor of Src and Syk tyrosine kinases	Targets cystine sites in NACHT domain to prevent ATPase and oligomerization	T2D, GA and CAPS	Preclinical	(170)
	Isoliquiritigenin	Licorice-derived polyphenol	Inhibits ATP-induced NLRP3 inflammasome activation	Diabetes Mellitus	Preclinical	(171)
	MCC950 (CP-456,773/CRID3)	Diarylsulfonylurea-containing small-molecule inhibitor	Blocks oligomerization by binding to NACHT domain and inhibiting ATPase activity	MS and PD	Preclinical	(172)
	C77	Benzoxazolone acetamide analog	Inhibits ATPase activity and ASC speck formation	Neurodegenerative diseases	Preclinical	(173)
	INF39	Acrylate derivative	Inhibits ATPase activity by covalent biding to NPLR3 NACHT domain	IBD	Preclinical	(174)
	OLT1117	β-sulfonyl nitrile compound	NACHT ATPase activity inhibition and NPLR3 activation blockade	Degenerative arthritis and CAPS	Phase 2	(175)
	BOT-4-one	Benzoxathiole derivative	Inhibits ATPase by alkylating NACHT domain and increase NLRP3 ubiquitination	Arthritis, dermatitis and peritonitis	Preclinical	(176)
	Compound 17 (YQ128)	Benzenesulfonamide derivative	Interfere with the NLRP3 inflammasome complex		Preclinical	(177)
	Miltefosine	Phosphocholine	Inhibition of NLRP3 inflammasome assembly	Cutaneous and visceral leishmaniasis	Approved	(178)
NLRP3 (indirectly)	Glyburide	Sulfonylurea	Inhibits K_ATP_ channel	T2D, gestational diabetes	Phase 4 (NCT00160485) (NCT00744965)	(143, 179)
	Gypenosides	Natural triterpene saponins	Inhibits ROS release and cytochrome c influx into cytoplasm	Diabetes Mellitus	Phase 2 (NCT02976766)	(180)
	Atorvastatin	Pentasubstituted pyrrole	Suppression of TLR4/MyD88/NF-κB pathway	Atherosclerosis and intracerebral hemorrhage	Phase 4 (NCT00590135) (NCT00645424)	(181, 182)
NLRP3, caspase-1, NF-κB	Parthenolide	Sesquiterpene lactone	Inhibits ATPase activity of NLRP3 Alkylates Cys residues of caspase 1 Inhibits activation of NF-κB by inhibiting IκB	Cystic fibrosis, leukemia and breast cancer	Phase 3 (NCT00640614)	(183–185)
NLRP3, caspase 1	Ginsenosides	Natural steroid glycosides and triterpene saponins	Inhibition of NLRP3 activation and caspase-1 activity	Diabetes mellitus	Phase 4 (NCT04167449) (NCT02204826)	(186, 187)
NLPR3, NF-κB	Bay 11-7082	Phenyl vinyl sulfone	Inhibits ATPase activity, probably through alkylation of Cys residues. Inhibition of NF-κB by blocking IKKβ		Preclinical	(188)
NLRP3 and downstream pathway	JC124	Benzenesulfonamide derivative	Inhibits NLRP3 without affecting ATPase activity Downregulates expression of NLRP3, ASC, pro-caspase-1, pro-IL-1β, TNF-α and iNOS	AD, TBI, AMI	Preclinical	(189–191)
ASC	JC171	Hydroxyl sulfonamide analogue	Blocks the recruitment of ASC during NLRP3 activation	MS	Preclinical	(192)
	IC100	Monoclonal antibody	Antibody-mediated neutralization of ASC	MS	Preclinical	(193)
Caspase-1	Ac-YVAD-CMK	Tetrapeptide	Potent and selective inhibition of caspase 1	Acute gastric injury, esophagitis and esophageal cancer	Preclinical	(194, 195)
	Belnacasan (VX-765)	Small molecule inhibitor	Potent and selective inhibition of caspase 1	Ischemia-associated BBB dysfunction	Phase 2 (NCT05164120)	(196)
NEK7	Licochalcone B	Flavonoid	Binds to NEK7 and prevents interaction with NLRP3	Septic shock, NASH, and peritonitis	Preclinical	(197)
IL-1β	Canakinumab	Monoclonal antibody	Binds to soluble human IL-1β and neutralizes the biological function	CAPS	Approved	(198)
	Anakinra	Recombinant human IL-1Ra	Binds to the IL-1R, competing with and inhibiting the activity of IL-1α and IL-1β	RA, deficiency of interleukin-1 antagonist and NOMID	Approved	(199)

MS, multiple sclerosis; PD, Parkinson´s disease; T2D, type 2 diabetes; GA, gouty arthritis; CAPS, cryopyrin-associated periodic syndrome; C172, analog of CFTR(inh)-172; MNS, 3;4-methylenedioxy-β-nitrostyrene; IBD, inflammatory bowel disease; IKKβ, Inhibitor of nuclear factor κ-B kinase subunit β; TNF-α, tumor necrosis factor α; iNOS, inducible nitric oxide synthase; AD, Alzheimer´s disease; TBI, traumatic brain injury; t53433efvAMI, acute myocardial infarction; K_ATP_ channel, ATP-sensitive K+ channels; NASH, non-alcoholic steatohepatitis; IL-1Ra, IL-1 receptor antagonist; RA, rheumatoid arthritis; NOMID, neonatal-onset multisystem inflammatory disease.

## 6 Conclusions

The interplay between the innate immune system, inflammation, metabolism, and metabolic diseases has revealed a complex and intertwined relationship. Inflammasomes, particularly the NLRP3 inflammasome, have emerged as critical mediators linking these processes together. The activation of inflammasomes triggers the release of proinflammatory cytokines IL-1β and IL-18 and the cell death by pyroptosis. The activation of NLRP3 inflammasome is under complex regulation involving multiple signaling networks. Mitochondrial dysfunction-mtROS production as well as metabolic signaling link the bioenergetics and metabolism status of the cell with cell death by pyroptosis, inflammation and innate immune functions through NLRP3 inflammasome activation.

Future studies should focus on elucidating the specific mechanisms by which inflammasomes contribute to metabolic dysfunction, identifying potential therapeutic targets within the inflammasome pathway, and developing interventions to modulate inflammasome activation and subsequent inflammation in metabolic diseases, and ultimately, improve patient outcomes and quality of life.

## Author contributions

All authors listed have made a substantial, direct, and intellectual contribution to the work and approved it for publication.
